# Foundations of Emergency Medicine: Application of a Flipped-Classroom Curriculum for Advanced Practice Clinician Education

**DOI:** 10.5811/westjem.42231

**Published:** 2025-09-12

**Authors:** Steven Lindsey, Tim P. Moran, Meredith A. Stauch, Alexis L. Lynch, Jordan Leumas, Kristen Grabow Moore

**Affiliations:** * Emory University, Department of Emergency Medicine, Atlanta, Georgia; † Emory University School of Medicine, Atlanta, Georgia; ‡ Wellstar Kennestone Regional Medical Center, Emergency Medicine Residency Program, Marietta, Georgia

## Abstract

**Introduction:**

Advanced practice clinician (APC) presence has increased in emergency departments (ED), leading to increased exposure to higher acuity patient conditions. Relatively few APCs have completed formalized postgraduate emergency medicine (EM)-specific training, creating uncertainty around how well prepared APCs are in identifying and treating life-threatening conditions. Foundations of Emergency Medicine (FoEM) offers free open-access curricula, including Foundations I (F1), a flipped-classroom course targeting fundamental knowledge for resident physicians. We sought to use F1 for APC learners to improve their knowledge in identifying and treating emergent conditions.

**Methods:**

In our single-center study, 23 APC postgraduate learners (17 nurse practitioners and 6 physician assistants) completed the F1 course between 2020–2021. The F1 course consisted of 23 virtual meetings led by faculty and senior residents, each lasting one hour. The APCs were asked to review vetted asynchronous resources for a recommended two hours before participating in small group, case-based learning sessions involving real-time feedback, curated teaching points, and paired online assessments. Immediately before and following F1 course implementation, participants completed a 50-question multiple-choice test and attitudes survey to quantify knowledge acquisition and evaluate the course. We evaluated change in knowledge scores using a Friedman test. Changes in self-assessed knowledge were evaluated using mixed-effects ordinal logistic regression.

**Results:**

Knowledge assessments showed APCs universally improved from the pre-course test (median score 23, 46%, IQR 20–26) to the post-course test (median score 33, 66%, IQR 31–37; adjusted P < .001). The APC self-assessments revealed improved overall EM knowledge (adjusted P = .02), yet respondents also reported an increased likelihood of seeking attending physician help (adjusted P < .001). Overall, 96% were satisfied with the course, 100% agreed that the course difficulty was appropriate, and 79% believed the course improved their performance in a clinical setting.

**Conclusion:**

Implementation of the Foundations of Emergency Medicine, Foundations 1, curriculum was associated with increased classroom knowledge and self-assessed overall knowledge in EM among advanced practice clinicians, with high learner satisfaction in the course. Along with knowledge improvement, APCs also reported increased likelihood to seek guidance from an attending physician. These data form the basis for the use of FoEM in the APC learner population.

## INTRODUCTION

Advanced practice clinicians (APC), including nurse practitioners (NP) and physician assistants (PA), have a significant presence in emergency medicine (EM). It is estimated that over 13,000 PAs and 26,000 NPs currently practice in acute care settings,^1^,^2^ with over 75% of emergency departments (ED) having APC representation.^3^,^4^ The APCs are involved in at least 20% of all ED visits, and the proportion of billing for higher acuity conditions by APCs is expanding over time,^5^–^7^ as APCs may work in higher acuity areas under direct physician supervision.^8^,^9^

Although some APCs care for patients with higher acuity pathologies in the ED,^7^ a relatively small proportion have completed formalized postgraduate training in EM, with 10% of PAs and 21% of NPs having received such training.^2^,^10^ The American Academy of Emergency Nurse Practitioners and the Society of Emergency Medicine Physician Assistants acknowledge the importance of APCs being able to identify and manage high-acuity conditions.^11^,^12^ Despite this, no consistent standardized approach exists to address this gap in EM-specific training.^13^,^14^

Foundations of Emergency Medicine (FoEM) is a free, open-access curriculum that offers standardized, level-specific core content. It is widely used in EM resident education with demonstrated success in improving knowledge acquisition.^15^,^16^ Foundations I (F1) is a longitudinal, flipped-classroom course developed for first-year resident physicians and provides systems-based review of fundamental EM knowledge. We sought to address the potential gap in APC EM-specific education and improve APC knowledge of higher acuity pathologies by implementing the F1 course.

## METHODS

### Study Population and Design

For our single-site study from October 2020–June 2021, APCs were included if they practiced at a large, urban, county, primarily adult hospital in Atlanta, Georgia, and enrolled in the FoEM F1 course. While course enrollment was mandatory to work in the higher acuity areas of the ED, study participation was voluntary. All APCs chose to participate in the course. Using a flipped-classroom model, APCs were asked to spend two hours reviewing vetted, multimodal, asynchronous study materials prior to each of the 23 didactic sessions. Implementation guidelines and curricular resources can be found on the FoEM website.^17^,^18^ The topics included all F1 content apart from trauma- and pediatric-related modules, which are rarely seen in the intended area for the APCs after course completion (Appendix 1). During one-hour virtual active learning sessions, participants collaboratively worked through case-based scenarios in small groups guided by faculty or upper-level EM resident group leaders. Instructors provided feedback on simulated clinical care and reviewed salient learning points, focusing on recognition, stabilization, evaluation, and treatment of higher acuity pathologies. While asynchronous studying was not tracked, APCs were required to attend 75% of sessions for course completion.

**Population Health Research Capsule**

What do we already know about this issue?

*Advanced practice clinicians (APC) see a significant number of patients, some of whom may have life-threatening conditions, yet their training curricula vary*.

What was the research question?

*Does the implementation of the Foundations of Emergency Medicine (FoEM) F1 course improve APC knowledge in emergent conditions?*

What was the major finding of the study?

*Knowledge improved from the pre-course (median [IQR] 23/50, 46%, [20–26]) to the post-course test (median [IQR] 33/50, 66%, [31–37]; P < .001)*.

How does this improve population health?

*The FoEM F1 curriculum improved APC knowledge of life-threatening conditions, while also increasing the likelihood of seeking an attending physician for guidance*.

To assess knowledge acquisition, study participants completed the same 50-question multiple-choice assessment pre- and post-course. Test items were curated by the two course directors (SL and KM) who referenced course objectives and selected, with permission, from assessment items that were developed and validated by Rosh Review specifically for use with FoEM courses. The APCs additionally completed a knowledge, attitudes, and practices (KAP) survey at the beginning and completion of the course (Appendix 2). The KAP survey was developed according to best practices in survey design.^19^,^20^ All survey items were piloted prior to administration. The post-course survey also included APCs’ impression of the course but was otherwise identical to the pre-course survey. This study was deemed exempt by the institutional review board of Emory University.

### Statistical Analysis

We described categorical variables using frequencies and percentages. Continuous and scale variables were described using medians and interquartile (IQR) ranges. We evaluated change in knowledge scores between the pre-course and post-course test sessions using the Friedman repeated-measures rank-order ANOVA. Ordinal self-assessment variables were evaluated using a mixed-effects ordinal logistic regression. We used mixed effects to account for multiple responses from individual study participants. To avoid inflated false positives due to multiple testing, we adjusted P values using the method developed by Benjamini and Yekutieli.^24^ Statistical analyses were conducted using R v4 (The R Foundation for Statistical Computing, Vienna, Austria).

## RESULTS

A total of 23 APCs enrolled, with a median age of 37 (IQR 33–40) years and with the majority identifying as female (74%) (Table 1). Learners were primarily family NPs (48%), followed by PAs (26%) and emergency NPs (22%), with a median of five years (IQR 3–6) of postgraduate experience in EM. A small proportion reported completing formalized postgraduate training in EM (13%).

**Figure 1:**
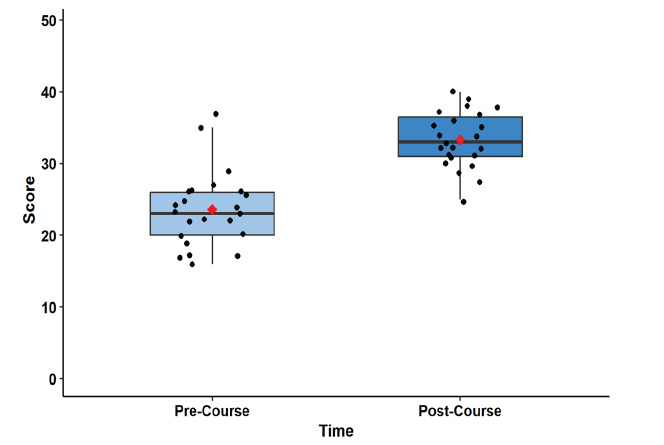
Objective knowledge acquisition of advanced practice clinicians before and after the Foundations of Emergency Medicine Foundations I course.

**Figure 2:**
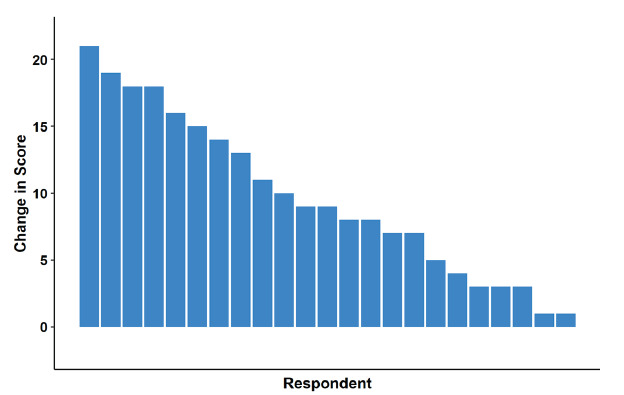
Change in individual knowledge test scores of advanced practice clinicians before and after Foundations of Emergency Medicine Foundations I course.

Knowledge assessments for the APCs significantly improved from the pre-course test to post-course test (P < .001, adjusted P < .001, Figure 1, Appendix 3), with a standardized median difference of 1.8 (95% CI 1.0–2.6). The pre-course test had a median (IQR) score of 23/50 (20–26) and the post-course test a median (IQR) score of 33/50 (31–37), with all APCs demonstrating improvement in their scores (Figure 2).

**Figure 3:**
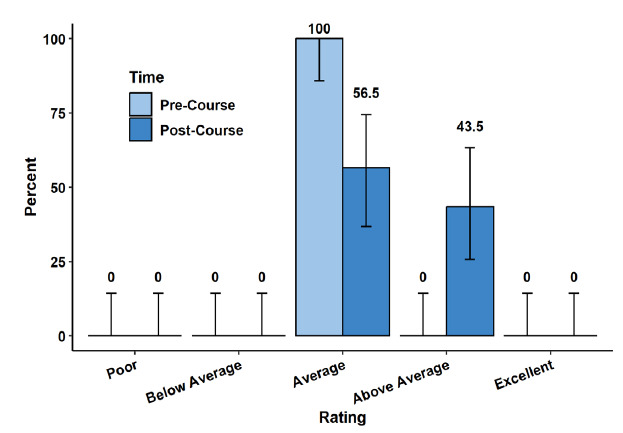
Self-rated knowledge in emergency medicine of advanced practice clinicians prior to and following Foundations of Emergency Medicine Foundations I Course.

With respect to self-assessed overall knowledge in EM, 100% of participants reported average knowledge prior to the course, compared to 43.5% above-average knowledge following the completion of the course (P = .01, adjusted P = .02) (Figure 3).

Because the units of educational assessments are often specific to the assessment, the effects of an intervention are often expressed as standardized mean or median differences.^21^–^23^ We present the mean and median difference in knowledge assessment, the standardized mean and median differences, and 95% CI. 21–23 The CIs were computed using bias-corrected and accelerated bootstrap resamples (100,000 resamples). As a rough rule, standardized differences of 0.2 are considered small, 0.5 are considered medium, and 0.8 are considered large.

Results from the KAP survey (Appendix 4) revealed significant improvement in APCs’ perceived ability to communicate with consultants (P < .001, adjusted P = .001) when caring for Emergency Severity Index (ESI) level 2 patients. Participants were significantly more likely to approach an attending physician to seek help with medical decision-making (P < .001, adjusted P < .001). No significant change was observed with APCs’ self-assessed ability to stabilize ESI level 2 patients in the ED (P = .03, adjusted P = .09) or APCs’ perceived ability to rule out life-threatening illnesses (P = .03, adjusted P = .09).

**Figure 4:**
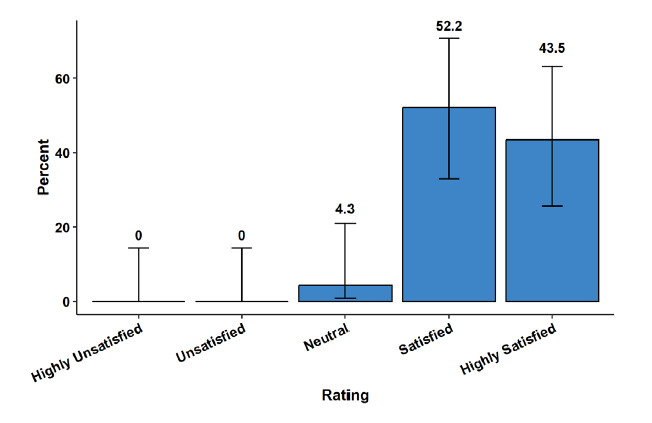
Advanced practice clinicians’ rating of overall satisfaction with the Foundations of Emergency Medicine Foundations I course.

In the post-course survey, 22/23 (96%) of APCs reported being either satisfied (52%) or highly satisfied (44%) with the course, while only 1/23 (4%) was neutral (Figure 4). When asked about the F1 course content being appropriate for the APCs’ level of learning, 23/23 (100%) responded as either agree (30%) or strongly agree (70%). The APCs either agreed (44%) or strongly agreed (35%) that the course improved their ability to manage high-acuity patients in the clinical environment. Only 5/23 were neutral, and no respondents disagreed.

## DISCUSSION

Advanced practice clinicians represent a sizable portion of the EM workforce^3^,^5^ and can work in both triage and variable acuity settings in the ED,^8^,^9^ making it of paramount importance that they recognize patients with more subtle presentations of higher acuity illnesses or potential life-threatening conditions. Our study demonstrated significant improvement in APC objective and self-assessed knowledge acquisition, along with gains in several facets of caring for higher acuity patients following implementation of the FoEM F1 course. Although the post-course multiple-choice scores were still low on average, there was significant improvement from the pre-course exam. Most notably, our findings have a high median standardized difference change of 1.8, reflecting a sizable improvement in exam scores. (See Appendix 3 for more explanation.) Our findings mirror those observed for electrocardiogram interpretation in the same cohort, namely the likelihood of approaching a physician for guidance, self-assessed knowledge improvement, and objective knowledge acquisition.^25^

It is possible that some of these knowledge gains were due to clinical experience and exposure; however, the curriculum was only nine months long and the APCs as individuals were infrequently in higher acuity areas of the ED over that time. While we are encouraged by the knowledge gained by the APCs in the F1 course, we recognize that classroom knowledge may not always translate to a meaningful change in practice. Our study does, however, provide a solid base on which to investigate the impact of F1 on clinical practice.

As is the case in some ED settings, APCs may not have immediate access to physician guidance.^26^ We advocate for direct physician supervision of APCs caring for higher acuity patients, and our study revealed that despite recognized knowledge gains, our APCs had increased likelihood of seeking guidance from an attending. We hypothesize that this may reflect better clinical understanding and more open lines of communication, although this would need better elucidation in future studies.

Improvement in APC knowledge and confidence in caring for subtle or overtly high-acuity ED patients is of great importance. To achieve this, APC learners must be motivated to engage in continuing education; thus, the delivery of educational material matters a great deal. The APCswho enrolled in our FoEM F1 course were overwhelmingly satisfied with the course and universally felt the content was appropriate for their level of training. Use of the case-based curriculum is in line with recommendations for APC education^27^ and translated to high engagement and knowledge acquisition.

## LIMITATIONS

Our study included a small sample size with a relatively homogeneous study population. Additionally, our study cohort did not fully reflect the 2:1 distribution of NPs and PAs practicing in acute care settings, with our group having only 26% PA representation. Due to the inherent differences in self-reported knowledge and objective tested knowledge, we chose not to attempt to correlate the two, instead focusing on them as individual findings in the study. Finally, our study did not include a control group with access to the curriculum, thereby allowing for the possibility of test/retest effects, although we believe this effect to have been small.^28^

## CONCLUSION

Foundations of Emergency Medicine offers a ready-made and well-received, case-based interactive learning modality for advanced practice clinicians in EM. By exposing our APC learners to the Foundations 1 course, our study demonstrated significant knowledge and confidence gains, along with a greater willingness to seek guidance from attending physicians. Future directions call for implementation and analysis of the curriculum in other EM APC learners, in larger cohorts, in different practice settings, and with more patient-oriented outcomes with the goal of a more representative reflection of validity across all EM-practicing APCs.

## Supplementary Information









## Figures and Tables

**Table 1:** Demographic characteristics of advanced practice clinicians enrolled in the Foundations of Emergency Medicine Foundations I Course, October 2020–June 2021.

Characteristic	Value N = 23
Age, Median (IQR)*	37 (33–40)
Gender, n (%)	
Female	17 (74%)
Male	6 (26%)
Certification, n (%)	
Adult Gerontology Nurse Practitioner	1 (4%)
Family Nurse Practitioner	11 (48%)
Family/Emergency Nurse Practitioner	5 (22%)
Physician Assistant	6 (26%)
Postgraduate Experience in Emergency Medicine, Median (IQR), years	5 (3–6)
Completed Emergency Medicine postgraduate training program, n (%)	3 (13%)

*IQR*, interquartile range.
