# Supplementary material for: Foundations of Emergency Medicine: Application of a Flipped-Classroom Curriculum for Advanced Practice Clinician Education
Source: West J Emerg Med. 2025 Sep 12;26(5):1226–31. doi: 10.5811/westjem.42231 (PMC12591618; doi:10.5811/westjem.42231)
Supplement: Supplementary file 1 [file wjem-26-1226-s001.docx]

**Appendix 1. Advanced Practice Provider Foundations of Emergency Medicine Foundations I Curriculum.**

| Unit | General Topics |
| --- | --- |
| GI I | GI Bleeding, Esophageal and Stomach Disorders |
| GI II | Acute Abdomen, Anorectal |
| GI III | Biliary, Liver, GI Infections |
| Pulm I | Non-infectious Pulmonary Disease |
| Pulm II | Infectious Pulmonary Disease |
| Cards I | Dysrhythmias |
| Cards II | ACS, CHF |
| Cards III | Valvular disease, Carditis, Syncope |
| Vascular | Dissection, Aneurysm, DVT & HTN |
| HEENT | Eye, Ear, Nose & Throat Emergencies |
| ID | Infectious Emergencies |
| Neuro I | Seizure, Coma, AMS, Vertigo, Headache, Meningitis |
| Neuro II | Stroke, Neuromuscular, Neuropathy, Spinal Cord |
| Tox I | Toxidromes and Poisoning I |
| Tox II | Toxidromes and Poisoning II |
| Environmental | Environmental Exposures |
| Renal / Male GU | Renal and Male Genitourinary Disorders |
| GYN | Ovarian and Uterine Disease, Gyn Infection |
| OB | Pregnancy Emergencies |
| Endo / Metabolic | Endocrine, Metabolic and Nutritional Disorders |
| Heme / Onc | Hematologic Emergencies, Emergent Complications of Malignancy |
| Psych | Psychiatric Emergencies |
| Immuno / Derm | Immune System Disorders, Skin Emergencies |
