# Supplementary material for: Foundations of Emergency Medicine: Application of a Flipped-Classroom Curriculum for Advanced Practice Clinician Education
Source: West J Emerg Med. 2025 Sep 12;26(5):1226–31. doi: 10.5811/westjem.42231 (PMC12591618; doi:10.5811/westjem.42231)
Supplement: Supplementary file 2 [file wjem-26-1226-s002.pdf]

# APP Foundations Course Survey (Grady-Post)

Thank you for taking the time to complete this survey.
We are asking for a unique identifier at the beginning of the survey for research purposes, but the survey is still completely anonymous, so please answer each question as honestly as possible.
The survey should take no more than 5-10 minutes.
Please note, several questions refer to ESI levels. If you are unfamiliar with this, please see below.

* Indicates required question

1. Please create your unique identifier based on this method: First letter of the city you grew up in (-) day of your birth in two numbers (-) childhood phone number area code. For example, the city of Gardner, the 24th of June, and 913-856-8937 would be answered as G-24-913. *

## ESI (Emergency Severity Index) levels

| | | | |
|---|---|---|---|
| Category 1 (RED) | Seen Immediately | Life Threatening Conditions | Cardiac/Respiratory Arrest<br>Immediate risk of airway, respiratory rate < 10/min, Extreme Respiratory Distress.<br>BP less than 80 in adult.<br>Severe shock in child/infant<br>GCS scale less than 9<br>Prolonged seizure<br>IV overdose<br>Severe behavioral disorder |
| Category 2 (ORANGE) | Seen within 10 minutes | Imminently life threatening, time sensitive treatment needed, or Severe pain. | Airway risk (stridor)<br>Circulatory Compromise (HR less than 50 or greater than 150, Hypotension, severe blood loss, poor perfusion). Chest pain likely cardiac related<br>Suspected sepsis, Febrile Neutropenia, Fever with lethargy<br>Acute Stroke<br>GCS less than 13<br>Suspected Testicular Torsion<br>High Risk History (toxic ingestion, venomous bite, pain suggesting PE, AAA, ectopic pregnancy. |
| Category 3 (GREEN) | Seen within 30 minutes | Potentially life threatening, situational urgency, or severe pain | Severe Hypertension, Moderate blood loss<br>Moderate Shortness of breath<br>Vomiting<br>Dehydration<br>Seizure (post ictal),<br>Head Injury with LOC (now alert)<br>Physiologically stable suspected sepsis<br>Severe pain<br>Limb injury consisting of limb deformity or severe laceration, altered sensation, absent pulse.<br>Potential child abuse<br>Behavioral/Psychiatric patient very distressed, risk of self-harm, potentially aggressive. |
| Category 4 (BLUE) | Seen within 60 minutes | Potentially serious condition, situational urgency or complex case | Mild Hemorrhage<br>Foreign Body Aspiration without respiratory distress<br>Chest injury without rib pain or respiratory distress<br>Minor head injury without LOC<br>Moderate pain<br>Vomiting or diarrhea without dehydration<br>Inflammation or foreign body in eye without vision changes<br>Minor limb trauma (ankle sprain, fracture, uncomplicated laceration with normal vital signs)<br>Swollen, erythematous joint<br>Semi Urgent mental health problems with no immediate risk to personnel. |
| Category 5 (white) | Seen within 120 minutes | Less urgent or Clinical-Administrative problems | Minimal pain with no risk factors<br>Low risk history<br>Minor symptoms of illness<br>Minor symptoms of low risk condition<br>Abrasions or minor laceration<br>Scheduled revisit<br>Immunizations<br>Patient with chronic psychiatric symptoms in social crisis. |

## Foundations Course Questions

2. Please rate your overall satisfaction with the Foundations-I APP Course.

*Mark only one oval.*

- Highly satisfied
- Satisfied
- Neutral
- Unsatisfied
- Highly unsatisfied

3. Foundations-I APP course content was appropriate for my level of learning.

*Mark only one oval.*

- Strongly agree
- Agree
- Neutral
- Disagree
- Strongly disagree

4. The Foundations-I APP course has improved my ability to manage high-acuity patients in the clinical environment.

*Mark only one oval.*

- Strongly agree
- Agree
- Neutral
- Disagree
- Strongly Disagree

5. What did you like about the Foundations-I APP course?

6. How can we improve the Foundations-I APP course?

Clinical confidence and practices questions

7. How would you rate your knowledge of emergency care in the following areas: *

*Mark only one oval per row.*

| | Poor | Below average | Average | Above average | Excellent |
|---|---|---|---|---|---|
| **Gastrointestinal diseases** | ◯ | ◯ | ◯ | ◯ | ◯ |
| **Respiratory diseases** | ◯ | ◯ | ◯ | ◯ | ◯ |
| **Cardiovascular diseases** | ◯ | ◯ | ◯ | ◯ | ◯ |
| **Ophthalmologic diseases** | ◯ | ◯ | ◯ | ◯ | ◯ |
| **Ear, Nose, Throat diseases** | ◯ | ◯ | ◯ | ◯ | ◯ |
| **Infectious Disease** | ◯ | ◯ | ◯ | ◯ | ◯ |
| **Neurologic diseases** | ◯ | ◯ | ◯ | ◯ | ◯ |
| **Toxicologic diseases** | ◯ | ◯ | ◯ | ◯ | ◯ |
| **Environmental diseases** | ◯ | ◯ | ◯ | ◯ | ◯ |

8. How would you rate your knowledge of emergency care in the following areas: *

*Mark only one oval per row.*

| | Poor | Below average | Average | Above average | Excellent |
|---|---|---|---|---|---|
| **Renal/ Genitourinary diseases** | ⬭ | ⬭ | ⬭ | ⬭ | ⬭ |
| **Obstetrical/ Gynecologic diseases** | ⬭ | ⬭ | ⬭ | ⬭ | ⬭ |
| **Endocrine/ Metabolic diseases** | ⬭ | ⬭ | ⬭ | ⬭ | ⬭ |
| **Hematologic/ Oncologic diseases** | ⬭ | ⬭ | ⬭ | ⬭ | ⬭ |
| **Psychiatric diseases** | ⬭ | ⬭ | ⬭ | ⬭ | ⬭ |
| **Immunologic diseases** | ⬭ | ⬭ | ⬭ | ⬭ | ⬭ |
| **Dermatologic diseases** | ⬭ | ⬭ | ⬭ | ⬭ | ⬭ |
| **EKG interpretation** | ⬭ | ⬭ | ⬭ | ⬭ | ⬭ |
| **Overall medical knowledge** | ⬭ | ⬭ | ⬭ | ⬭ | ⬭ |

9. In general, how confident are you in INDEPENDENTLY caring for the following Emergency Severity Index (ESI) level patients while working clinically in the emergency department? *

*Mark only one oval per row.*

| | Not confident | Somewhat confident | Confident | Very confident |
|---|---|---|---|---|
| ESI Level 1 (Resuscitation) | ◯ | ◯ | ◯ | ◯ |
| ESI Level 2 (Emergent) | ◯ | ◯ | ◯ | ◯ |
| ESI Level 3 (Urgent) | ◯ | ◯ | ◯ | ◯ |
| ESI Level 4 (Less Urgent) | ◯ | ◯ | ◯ | ◯ |
| ESI Level 5 (Non-urgent) | ◯ | ◯ | ◯ | ◯ |

10. If given the choice between picking up an ESI Level 4 (Less Urgent) or an ESI Level 2 (Emergent) patient while working a shift in the emergency department, which are you most likely to choose?

*Mark only one oval.*

| | 1 | 2 | 3 | 4 | 5 | |
|---|---|---|---|---|---|---|
| Mor | ◯ | ◯ | ◯ | ◯ | ◯ | More likely ESI 2 patient |

11. If given the choice between picking up an ESI Level 3 (Urgent) or an ESI Level 1 (Resuscitation) patient while working a shift in the emergency department, which are you most likely to choose? *

*Mark only one oval.*

| | 1 | 2 | 3 | 4 | 5 | |
|---|---|---|---|---|---|---|
| Mor | ⬭ | ⬭ | ⬭ | ⬭ | ⬭ | More likely ESI 1 patient |

12. When caring for an ESI Level 2 (Emergent) patient, how confident are you in INDEPENDENTLY performing the following skills: *

*Mark only one oval per row.*

| | Not confident | Somewhat Confident | Confident | Very Confident |
|---|---|---|---|---|
| **Providing emergent stabilization** | ◯ | ◯ | ◯ | ◯ |
| **Completing a focused history and physical** | ◯ | ◯ | ◯ | ◯ |
| **Deciding on initial orders for evaluation (e.g., labs, imaging)** | ◯ | ◯ | ◯ | ◯ |
| **Deciding on initial orders for management (e.g., medications, procedures)** | ◯ | ◯ | ◯ | ◯ |
| **Ruling-out life-threatening illness** | ◯ | ◯ | ◯ | ◯ |
| **Identifying the most likely diagnosis** | ◯ | ◯ | ◯ | ◯ |
| **Identifying the need for subspecialist consultation** | ◯ | ◯ | ◯ | ◯ |
| **Communicating with a consultant about a patient** | ◯ | ◯ | ◯ | ◯ |
| Deciding on the | ◯ | ◯ | ◯ | ◯ |

Deciding on the specific disposition for a patient (e.g., discharge, admit to floor, admit to ICU, etc.)

specific disposition for a patient (e.g., discharge, admit to floor, admit to ICU, etc.)

13. When you are uncertain about medical decision-making for a patient in your care, how likely are you to ask for guidance from: *

*Mark only one oval per row.*

| | Very Unlikely | Unlikely | Likely | Very Likely |
|---|---|---|---|---|
| **Another Advanced Practice Provider** | ◯ | ◯ | ◯ | ◯ |
| **A Resident Physician** | ◯ | ◯ | ◯ | ◯ |
| **An Attending Physician** | ◯ | ◯ | ◯ | ◯ |

14. How comfortable do you feel asking an attending physician for guidance regarding medical decision-making for a patient in your care? *

*Mark only one oval.*

| | 1 | 2 | 3 | 4 | 5 | |
|---|---|---|---|---|---|---|
| Very | ◯ | ◯ | ◯ | ◯ | ◯ | Very Comfortable |

15. How comfortable do you feel asking an attending physician for feedback about your clinical performance during or after a shift in the emergency department? *

*Mark only one oval.*

| | 1 | 2 | 3 | 4 | 5 | |
|---|---|---|---|---|---|---|
| Very | ◯ | ◯ | ◯ | ◯ | ◯ | Very Comfortable |

16. How often do you ask an attending physician for feedback about your clinical performance during or after a shift in the emergency department? *

*Mark only one oval.*

| | 1 | 2 | 3 | 4 | 5 | |
|---|---|---|---|---|---|---|
| Alm | ◯ | ◯ | ◯ | ◯ | ◯ | Almost Always |

17. How often do you reference a Free Open Access Medical (FOAM) education resource (e.g., a medical blog) when trying to answer a clinical question? *

*Mark only one oval.*

| | 1 | 2 | 3 | 4 | 5 | |
|---|---|---|---|---|---|---|
| Not | ◯ | ◯ | ◯ | ◯ | ◯ | Very Often |

18. What FOAM education resources (e.g., medical blogs) do you frequently reference to answer a clinical question? *

______________________________

19. Of the FOAM resources that you reference, how confident are you in the reliability of the clinical information? *

*Mark only one oval.*

| | 1 | 2 | 3 | 4 | 5 | |
|---|---|---|---|---|---|---|
| Not | ◯ | ◯ | ◯ | ◯ | ◯ | Very confident |

This content is neither created nor endorsed by Google.

Google Forms
