# Supplementary material for: Foundations of Emergency Medicine: Application of a Flipped-Classroom Curriculum for Advanced Practice Clinician Education
Source: West J Emerg Med. 2025 Sep 12;26(5):1226–31. doi: 10.5811/westjem.42231 (PMC12591618; doi:10.5811/westjem.42231)
Supplement: Supplementary file 3 [file wjem-26-1226-s003.docx]

**Appendix 3. Advanced Practice Provider Foundations of Emergency Medicine Foundations I Knowledge Assessment Analysis Results**

| **Metric** | **Pre-Test**  **(95% CI)** | **Post-Test**  **(95% CI)** | **Difference**  **(95% CI)** | **Standardized Difference**  **(95% CI)** | **p** | **p-adj** |
| --- | --- | --- | --- | --- | --- | --- |
| Mean % Correct | 47.2 (43.5–51.2) | 66.6 (63.7–69.5) | 19.4 (14.8–24.1) | 1.6 (1.2–2.1) | <.001 | <.001 |
| Median % Correct | 46 (44–52) | 66 (62–70) | 20 (18–22) | 1.8 (1.0–2.6) | <.001 | <.001 |

CI: Confidence Interval

Because the units of educational assessments are often specific to the assessment, the effects of an intervention are often expressed as standardized mean or median differences. ^21-23^ We present the mean and median difference in knowledge assessment, the standardized mean and median differences, and 95% confidence interval (95% CI). ^21-23^ CIs were computed using bias-corrected and accelerated bootstrap resamples (100,000 resamples). As a rough rule, standardized differences of 0.2 are considered small, 0.5 are considered medium, and 0.8 are considered large.
