# Supplementary material for: Foundations of Emergency Medicine: Application of a Flipped-Classroom Curriculum for Advanced Practice Clinician Education
Source: West J Emerg Med. 2025 Sep 12;26(5):1226–31. doi: 10.5811/westjem.42231 (PMC12591618; doi:10.5811/westjem.42231)
Supplement: Supplementary file 4 [file wjem-26-1226-s004.docx]

**Appendix 4. Advanced Practice Provider (APP) Self-Reported Knowledge, Attitudes, and Practices (KAP) Before and After Foundations of Emergency Medicine (FoEM) Foundations I Course.**

| **Item** | **Pretest**  **N (%)** | **Posttest**  **N (%)** | **OR**  **(95% CI)** | **p** | **p-adj** |
| --- | --- | --- | --- | --- | --- |
| **ESI 2 – Emergent Stabilization** |  |  | 2.75 (1.10-6.91) | .03 | .09 |
| Not Confident | 7 (30.4) | 2 (8.7) |  |  |  |
| Somewhat Confident | 7 (30.4) | 8 (34.8) |  |  |  |
| Confident | 9 (39.1) | 11 (47.8) |  |  |  |
| Very Confident | 0 (0) | 2 (8.7) |  |  |  |
| **ESI 2 – Ruling out life-threatening illness** |  |  | 2.84 (1.10-7.36) | .03 | .09 |
| Not Confident | 1 (4.3) | 0 (0) |  |  |  |
| Somewhat Confident | 14 (60.9) | 10 (43.5) |  |  |  |
| Confident | 8 (34.8) | 11 (47.8) |  |  |  |
| Very Confident | 0 (0) | 2 (8.7) |  |  |  |
| **ESI 2 - Communicating with Consultant** |  |  | 17.9 (3.85-83.6) | <.001 | .001 |
| Not Confident | 0 (0) | 0 (0) |  |  |  |
| Somewhat Confident | 6 (26.1) | 1 (4.3) |  |  |  |
| Confident | 16 (69.6) | 9 (39.1) |  |  |  |
| Very Confident | 1 (4.3) | 13 (56.5) |  |  |  |
| **Likelihood of approaching attending with help regarding medical decision** |  |  | 52 (8.5-317) | < .001 | <.001 |
| Very Unlikely | 0 (0) | 0 (0) |  |  |  |
| Unlikely | 3 (13) | 0 (0) |  |  |  |
| Likely | 20 (87) | 4 (17.4) |  |  |  |
| Very Likely | 0 (0) | 19 (82.6) |  |  |  |
| **Comfort asking attending for guidance** |  |  | 0.32 (0.10-0.97) | .04 | .13 |
| 1 | 0 (0) | 1 (4.3) |  |  |  |
| 2 | 0 (0) | 0 (0) |  |  |  |
| 3 | 0 (0) | 0 (0) |  |  |  |
| 4 | 4 (17.4) | 8 (34.8) |  |  |  |
| 5 | 19 (82.6) | 14 (60.9) |  |  |  |
| **Foundations was appropriate for my level** |  |  |  |  |  |
| Strongly Disagree | - | 0 (0) |  |  |  |
| Disagree | - | 0 (0) |  |  |  |
| Neutral | - | 0 (0) |  |  |  |
| Agree | - | 7 (30.4) |  |  |  |
| Strongly Agree | - | 16 (69.6) |  |  |  |
| **Foundations Course Improved my Ability to Manage High Acuity Patients** |  |  |  |  |  |
| Strongly Disagree | - | 0 (0) |  |  |  |
| Disagree | - | 0 (0) |  |  |  |
| Neutral | - | 5 (21.7) |  |  |  |
| Agree | - | 10 (43.5) |  |  |  |
| Strongly Agree | - | 8 (34.8) |  |  |  |
| **Overall Satisfaction with the Foundations Course** |  |  |  |  |  |
| Highly Unsatisfied | - | 0 (0) |  |  |  |
| Unsatisfied | - | 0 (0) |  |  |  |
| Neutral | - | 1 (4.3) |  |  |  |
| Satisfied | - | 12 (52.2) |  |  |  |
| Highly Satisfied | - | 10 (43.5) |  |  |  |

CI: Confidence Interval; ESI: Emergency Severity Index
